# The compliance of free folic acid supplements among pregnant women in rural areas of Northwestern China: The role of related knowledge

**DOI:** 10.3389/fpubh.2022.1079779

**Published:** 2023-01-09

**Authors:** Jie Yang, Zulihumaer Reheman, Yunjie Liu, Yuan Wang, Nan Wang, Jinbiao Ye, Yangyuan Li, Jingchun Nie

**Affiliations:** ^1^Center for Experimental Economics in Education, Shaanxi Normal University, Xi'an, China; ^2^Management, Strategy, and Entrepreneurship Department, College of Business, University of Houston-Victoria, Victoria, TX, United States

**Keywords:** folic acid supplementation, folic acid usage, knowledge, neural tube defects, folic acid policy, pregnancy, rural areas

## Abstract

**Background:**

High prevalence of neural tube defects remains one of the major threats to newborns in rural China. Folic acid supplementation before and during early pregnancy can effectively reduce the risk of neural tube defects. Despite the efforts of the free folic acid mass distribution, the actual usage of folic acid supplements was still suboptimal among rural pregnant women in China. The objective of this study is to investigate if and how knowledge can influence the picking up and intake of the free folic acid supplements distributed by the government.

**Methods:**

We collected survey data from 821 pregnant women in rural areas of Shaanxi, China, in March and December of 2021. Face-to-face interviews and questionnaire surveys were conducted with every participant. Univariate and multivariate logistic regression analyses were performed to test the relationship between knowledge and dependent variables.

**Results:**

Our study found that there were 76.4% of pregnant women would pick up folic acid supplements distributed by the government and only 44.5% of women would use folic acid before current pregnancy. Awareness of folic acid policy both affects the picking up (*OR*: 6.708, 95% *CI*: 4.672–9.632) and periconceptional intake (*OR*: 1.912, 95% *CI*:1.326–2.758) of folic acid supplements. Knowledge of health and nutrition in pregnancy showed no significant relationship with the picking up and periconceptional intake of folic acid supplements but was positively associated with the intake duration (*Coefficient*: 9.278, 95% *CI*: 2.966–15.591).

**Conclusion:**

Despite the relatively high level of picking up, the actual folic acid usage was not ideal among pregnant women in rural areas of China. Folic acid policy awareness was positively associated with the picking up and intake of folic acid before and during conception. Knowledge of health and nutrition about pregnancy was related to a longer duration of folic acid intake but had no impact on the picking up rate and periconceptional intake of folic acid supplements.

## 1. Introduction

Neural tube defects (NTDs) are one of the most common birth defects worldwide with a prevalence rate that varies from 0.5 to more than 10 per 1,000 pregnancies (1), which can lead to infant mortality and severe disability. In China, there are significant geographic and economic regional differences in the incidence of NTDs, which are manifested in the north being higher than the south, and the rural areas being higher than the urban areas (2). Several studies have documented that in rural areas of northern China, although the distribution of free folic acid supplements has been launched for several years, the prevalence of NTDs can still be as high as 31.5 per 10,000 births (3), which is much higher than in urban areas (4).

The incidence and recurrence of NTDs can be effectively prevented by daily supplementation with folic acid. Since the neural tube closes by day 28, taking folic acid supplementation preconceptionally as a preventative measure is especially important to reduce the risk of NTDs (5–9). The WHO has reported that sufficient folic acid supplementation can lower the incidence of NTDs by at least 47% in low and middle-income countries (10). Therefore, government officials in many countries took various measures and endeavored to promote adequate folic acid supplementation among reproductive-aged women since the 1990's, such as the mandatory food fortification programs adopted by many developed countries (11) and the iron-folic acid supplementation programs implemented in many developing countries (12–14). In 2009, the Chinese government launched a nationwide distribution of free folic acid supplementation for reproductive-aged women to prevent NTDs (15). Now, all reproductive-aged women (women preparing for pregnancy or within 3 months of pregnancy) in China can voluntarily pick up free folic acid pills as many as six bottles at once at the community health centers or village clinics with the registration of identity card.

However, improving the actual usage of folic acid supplements is still a worldwide challenge in preventing NTDs. Studies conducted in South Australia, India, Kenya, and Ethiopia showed that the compliance rate of folic acid usage was 40, 64.7, 32.7, and 73.2% respectively (13, 14, 16, 17). The dietary folate intake in China was suboptimal and varied by region (18). A large-scale survey conducted in Shaanxi province of China showed that 66. 4% of the respondents (63. 4% for rural and 72. 6% for urban) used folic acid before or during the pregnancy, but only 25. 3% of them used folic acid before pregnancy (19). Another study conducted in Shanxi province of China reported that 43.1% of women had picked up free folic acid but only 16.4% of them took folic acid supplements following the recommendations of WHO, which is to start taking folic acid supplements from at least 3 months before pregnancy to 3 months after pregnancy, plus taking the supplements for more than 8 days within 10 days (20). Other studies in China also reported relatively high picking-up rates of free folic acid supplements with low intake and compliance rates (21–24).

Unfortunately, such discrepancies between picking-up and the actual usage of folic acid supplements have not been thoroughly investigated. It remains unclear if the free folic acid supplements distributed were being used accurately, because we can never reduce the incidence of NTDs if the women did not intake the supplements they picked up from the local clinics. Then the bigger question arose here is that to what type of person, the free public health services can be most effective? To answer this question, Noor et al. (25) conducted an experiment to argue about the effectiveness of health products massively distributed freely against products being purchased. Their results indicated that only 43.6% of the children who received free nets distributed by the government would actually sleep under a net, much lower than the average usage proportion of 67.3%. But they also reported the heterogeneity of the effects and found that free nets were the most popular among the poorest. Following Noor's arguments, it is rational for us to assume that economically disadvantaged women will be more willing to take advantage of the free folic acid supplements distributed by the government. What are other potential correlated factors of the usage of free folic acid supplements?

Previous studies on the compliance of folic acid mainly focus on the intake of the supplements, and seldom have investigated the antecedences for picking up and the usage of free folic acid supplements in rural China. Previous studies indicated that the intake rate and periconceptional use of folic acid supplements are associated with the knowledge about folic acid (26–28) and sociodemographic factors such as age, education, unplanned pregnancy, pregnancy order, body mass index (BMI), and family asset (14, 17, 29, 30). Several studies pointed out that the lack of awareness of folic acid is one of the main factors related to the low intake rate of folic acid in China (22, 27, 31), but few of them included more in-depth analyses.

Therefore, this study aims to first describe the status quo of folic acid policy in rural areas of northwestern China in terms of the picking-up rate and intake rate. Secondly, this study is trying to explore if and how knowledge, in terms of the overall knowledge of health and nutrition, the correct time to use folic acid, as well as the awareness of folic acid policy can influence the picking up and intake rate. The findings of this study make important implications for decision-makers to optimize policy implementation for both China and other developing countries.

## 2. Materials and methods

### 2.1. Study population and sampling

We used the baseline data of a randomized controlled trial carried out by Shaanxi Normal University in rural areas of Shaanxi Province on maternal and infant health. Located in northwest China, rural areas of Shaanxi Province were reported to have a relatively lower prevalence of NTDs than the northern average with 18.5 per 10,000 pregnancies, however, the NTDs remain the first threat among the top four types of birth defects in these areas (32). We conducted two rounds of survey collection in March and December 2021 with the same questionnaire in the same sample sites.

We employed a multilevel cluster random sampling method to identify our potential participants. This project involved five prefectures of Shaanxi Province, and two counties were selected from each prefecture. The specific sampling procedures are as follows. First, all towns in the sample county were included except for the town where the county seat to form our sampling frame, then ten townships were randomly selected for the counties with more than ten townships; for counties with less than ten townships, all townships were included. Secondly, a list of all pregnant women was obtained with the help of the local health bureau in the sample towns. Considering the survey cost, towns with less than three samples were excluded. In total, the study included 79 townships in ten counties. Finally, ten pregnant women were randomly selected in each township to participate in the survey conducted in March. For towns with less than ten observations, all observations were included. In the survey conducted in December, we randomly selected five women who were pregnant after June in each township, and for towns with less than five observations, all observations were included. Due to ethical and practical considerations and to ensure that participants are indeed representative of the target population, the inclusion criteria of our participants were: (1) aged between 18-45 years; (2) had resided locally for ≥1 year; (3) within six months of pregnancy; and (4) had no history of mental illness and other serious diseases. Eventually, a total of 821 completed survey were obtained from pregnant women where 592 observations from March and 229 observations from December.

### 2.2. Survey method

We used a questionnaire survey method in our study, with trained enumerators conducting face-to-face interviews with every participant. The questionnaire includes questions on the sociodemographic characteristics, health, and mental health status of pregnant women, as well as questions regarding the knowledge about health and nutrition during pregnancy and postpartum.

A consent form with information about program objectives, procedure, potential risks, and benefits for participants, as well as privacy protection was distributed to each eligible participant before the face-to-face interview. To ensure accuracy and consistency during the procedure of data collection, the enumerators were trained intensively, followed by a pilot study with twenty participants prior to large-scale data collection. All interview questions were shown on a tablet and asked one by one by the enumerator while answers were recorded at the same time. Each interview was conducted alone to avoid interruptions from other family members.

### 2.3. Definition and measurement of variables

The dependent variables of this study include picking-up folic acid supplements, intake of folic acid supplements, and intake duration. Picking-up of folic acid supplements was defined as having picked up the free folic acid at community health centers or village clinics. Intake of folic acid supplements refers to the actual usage of free folic acid supplements before the current pregnancy. Intake duration was defined as the total number of days of taking folic acid before or during the current pregnancy.

Knowledge served as the independent variable of this study, which contains three sub-dimensions: the overall knowledge of health and nutrition, the correct time to use folic acid, as well as the awareness of the folic acid policy. The overall knowledge of health and nutrition was measured by 12 questions (6 questions on nutrition and 6 questions on health) on the knowledge about pregnancy. A correct answer for each question was scored as 1 and then summed up. We used standardized scores (z-scores) of the total scores for our analyses. Two categories of health and nutrition knowledge were included in this study, in terms of overall knowledge on nutrition such as the correct time to use folic acid, foods rich in iron, etc.; as well as the overall knowledge on health regarding physical and mental healthcare during pregnancy such as normal blood pressure range for pregnant women, correct way to deal with stress, etc. We also used standardized scores (z-scores) of the total scores of these two categories in our following statistical analyses. The second sub-dimension of knowledge, the correct time to use folic acid, referred to 3 months before conception to the first trimester of pregnancy. The last subdimension of knowledge, awareness of the folic acid policy, measures whether the pregnant woman was informed of the free folic acid distribution policy.

Sociodemographic characteristics include age, education, employment status, pregnancy order and intention, self-reported health status, whether the pregnant woman was living together with her husband, whether the family asset of the pregnant woman was in the lowest 25% among all of them, and whether her family was officially registered as poverty-stricken households in 2019. Considering that there are south-north differences in the plasma folate status (18) and the prevalence of NTDs (4), regions of the county where the pregnant woman was located were classified into north and south by Qinling Mountains. Body mass index (BMI; in weight (kg)/height(*m*)^2^) used in the study was calculated by the height and weight of pregnant women measured by us with the assistance of local nurses using unified standard weight scales and tape measures before the survey. Based on the literature and WHO classification, BMI was divided into three categories (<25 / 25≤BMI<30 / ≥30) (30, 33).

### 2.4. Statistical analysis

The data processing and statistical analyses in this study were performed using STATA 15.0 software. Categorial variables were summarized with frequency and percentage, while continuous variables were presented by the mean and standard deviation (*SD*) in descriptive analyses. Univariate and multivariate logistic regression analyses were performed to test the relationship between knowledge and dependent variables, in terms of picking-up and periconceptional intake of folic acid supplements. We also used multiple linear regression analyses to explore the relationship between variables and intake duration. The statistical level of significance was *P* < 0.05 and the outcomes of regression analyses were presented as odds ratios (*OR*s) or coefficients with their 95% confidence intervals (95% CIs).

## 3. Results

### 3.1. Characteristics of participants

Table 1 shows the basic demographic characteristics of the 821 pregnant women included in this study. The mean age of these respondents was 28.8 years (*SD* = 4.33). More than half (58.2%) of these women did not have high school education and the majority (73.8%) of them did not have a steady job at that time. 65.5% of them were expecting their first babies and 61.8% of these pregnancies were planned.

**Table 1:** Sociodemographic characteristics of study population.

**Variables**	**Distribution (*****n*** = **821)**	
	***N***	**(%)**
**Age**		
<28	356	(43.4)
≥28	465	(56.6)
**Years of education**		
≤ 9 years	478	(58.2)
>9 years	341	(41.5)
Missing	2	(0.3)
**Employment status**		
Unemployed	606	(73.8)
Employed	215	(26.2)
**Pregnancy order**		
First	538	(65.5)
Second or Higher	283	(34.5)
**Pregnancy intention**		
Intended	507	(61.8)
Unintended	314	(38.2)
**Body mass index**		
BMI <25	404	(49.2)
25≤BMI <30	314	(38.3)
BMI≥30	103	(12.5)
**Self-reported health status**		
Good, moderate, or poor	584	(71.1)
Excellent	237	(28.9)
**Living with husband**		
Yes	534	(65.0)
No	287	(35.0)
**Family asset in lowest 25%**		
Yes	196	(23.9)
No	625	(76.1)
**Officially registered as poverty-stricken household**		
Yes	114	(13.9)
No	707	(86.1)
**Region**		
South	474	(57.7)
North	347	(42.3)

### 3.2. Descriptive analyses of compliance of folic acid supplements and related knowledge

In Table 2, we described the status quo of all three aspect of folic acid compliance, including picking-up, intake, and intake duration. Among all the 821 women, 627 of them (76.4%) reported that they had picked up folic acid before or during pregnancy, and only 365(44.5%) of them used folic acid before their pregnancy. The mean of the total days they took folic acid pre-pregnancy or during pregnancy was 110.3 days (*SD* = 79.5).

**Table 2:** Descriptive analyses of compliance of free folic acid supplements and related knowledge.

**Variables**	***N*** **/ Mean**	**(%) / SD**
**Compliance of folic acid**		
Picking-up	627	(76.4)
Intake	365	(44.5)
Intake duration	110.3	79.5
**Related knowledge**		
Score of overall knowledge of health and nutrition	6.5	2.2
Knew correct time to take folic acid	658	(80.2)
Awareness of folic acid policy	602	(73.3)

In terms of health and nutrition knowledge, the mean score of their overall knowledge on health and nutrition was 6.5 (*SD* = 2.2). 80.2% of the respondents knew the correct time to use folic acid and 26.7% of them were still not aware of the free folic acid distribution policy.

### 3.3. Factors associated with picking up folic acid supplements

Results of the univariate and multivariate logistic regression analyses of factors related to the picking up of folic acid are shown in Table 3. It is obvious that the expectant women who were aware of the free folic acid distribution policy had 6.7 times higher odds of picking up (*OR*: 6.708, 95% *CI*: 4.672-9.632) free folic acid supplements than their counterparts. Other variables showed no significant association with the picking up of folic acid both in univariate and multivariate analyses.

**Table 3:** Results of univariate and multivariate logistic regression analyses of variables related to the picking up of folic acid supplements.

**Dependent variable: picking-up of folic acid supplements**	**Univariate**		**Multivariate**	
	**OR (95% CI)**	***P*** **-value**	**OR (95% CI)**	***P*** **-value**
**Knowledge**				
Overall knowledge of health and nutrition (standardized score)	1.143 (0.974–1.342)	0.101	1.105 (0.886–1.379)	0.374
Knew correct time to take folic acid (1 = yes)	1.203 (0.812–1.782)	0.356	1.004 (0.624–1.617)	0.986
Awareness of folic acid policy (1 = yes)	6.427 (4.520–9.138)	<0.001	6.708 (4.672–9.632)	<0.001
**Characteristics of respondents**				
Age (<28 as reference)	1.053 (0.761–1.456)	0.756	0.998 (0.679–1.467)	0.992
Years of education (≤9 years as reference)	1.068 (0.769–1.483)	0.694	1.070 (0.693–1.652)	0.759
Employment status (1 = employed)	0.837 (0.585–1.199)	0.332	0.716 (0.473–1.084)	0.115
Pregnancy order (1 = first)	1.026 (0.731–1.441)	0.880	0.867 (0.576–1.304)	0.492
Pregnancy intention (1 = intended)	1.083 (0.779–1.506)	0.636	1.204 (0.833–1.740)	0.323
BMI (BMI < 25 as reference)	1.098 (0.795–1.516)	0.569		
25 ≤ BMI < 30	0.898 (0.646–1.248)	0.520	0.846 (0.570–1.257)	0.408
BMI ≥ 30	1.021 (0.627–1.664)	0.933	0.845 (0.479–1.493)	0.563
Self-reported health status (1 = excellent)	1.034 (0.724–1.477)	0.856	1.070 (0.711–1.611)	0.746
Living together with husband (1 = yes)	1.131 (0.809–1.581)	0.471	1.153 (0.790–1.684)	0.460
Family asset in lowest 25% (1 = yes)	1.012 (0.693–1.477)	0.952	1.176 (0.753–1.837)	0.477
Officially registered as poverty-stricken household (1 = yes)	0.688 (0.444–1.067)	0.095	0.642 (0.397–1.037)	0.070
Region (1 = north)	0.760 (0.550–1.051)	0.097	0.730 (0.506–1.053)	0.092

95% CI, 95% confidence intervals.

### 3.4. Factors associated with periconceptional intake of folic acid

Univariate analyses in Table 4 present that women who were aware of the free folic acid distribution policy (*OR*:1.604, 95% *CI*: 1.166–2.206), expecting their first child (*OR*:2.067, 95% *CI*: 1.543–2.768) or pregnant by intention (*OR*:9.558, 95% *CI*: 6.679–13.680) were more likely to take folic acid before conception. Multivariate analyses reported similar results and showed that pregnant women from poverty-stricken households had fewer chances of pre-pregnancy intake of folic acid (*OR*:0.615, 95% *CI*: 0.396–0.954) than their counterparts.

**Table 4:** Results of univariate and multivariate logistic regression analyses of variables related to the periconceptional intake of folic acid supplements.

**Dependent variable: periconceptional intake of folic acid supplements**	**Univariate**		**Multivariate**	
	**OR (95% CI)**	***P*** **-value**	**OR (95% CI)**	***P*** **-value**
**Knowledge**				
Overall knowledge of health and nutrition (standardized score)	1.119 (0.974–1.285)	0.113	1.052 (0.857–1.291)	0.630
Knew correct time to take folic acid (1 = yes)	1.263 (0.891–1.791)	0.189	1.023 (0.630–1.661)	0.927
Awareness of folic acid policy (1 = yes)	1.604 (1.166–2.206)	0.004	1.912 (1.326–2.758)	0.001
**Characteristics of respondents**				
Age (<28 as reference)	1.047 (0.793–1.382)	0.748	1.218 (0.857–1.732)	0.271
Years of education (≤9 years as reference)	1.288 (0.974–1.703)	0.076	1.069 (0.750–1.525)	0.712
Employment status (1 = employed)	1.037 (0.758–1.418)	0.821	0.844 (0.581–1.225)	0.372
Pregnancy order (1 = first)	2.067 (1.543–2.768)	<0.001	1.636 (1.135–2.358)	0.008
Pregnancy intention (1 = intended)	9.558 (6.679–13.680)	<0.001	9.742 (6.687–14.193)	<0.001
BMI (BMI <25 as reference)	1.091 (0.828–1.436)	0.537		
25 ≤ BMI < 30	0.927 (0.698–1.232)	0.603	0.944 (0.666–1.338)	0.746
BMI ≥ 30	0.965 (0.636–1.463)	0.867	1.023 (0.620–1.688)	0.928
Self-reported health status (1 = excellent)	1.145 (0.845–1.550)	0.383	1.030 (0.716–1.481)	0.875
Living together with husband (1 = yes)	1.260 (0.942–1.685)	0.119	1.017 (0.725–1.427)	0.923
Family asset in lowest 25% (1 = yes)	0.778 (0.562–1.079)	0.133	0.874 (0.588–1.299)	0.505
Officially registered as poverty-stricken household (1 = yes)	0.724 (0.482–1.087)	0.120	0.615 (0.396–0.954)	0.030
Region (1 = north)	1.123 (0.850–1.483)	0.415	1.135 (0.816–1.579)	0.453

95% CI, 95% confidence intervals.

### 3.5. Influencing factors of intake duration

From the multiple linear regression analysis results summarized in Table 5, we found that each standard deviation increase in the overall knowledge of health and nutrition during pregnancy can increase the duration of folic acid intake by 9.3 days (column 1). In order to figure out the separate impact of knowledge of nutrition and knowledge of health on the folic acid intake duration, we performed multiple linear regressions in column 2 and column 3. We can conclude from the results that both the knowledge of health (*Coefficient*: 5.362, 95% *CI*: 0.465–10.259) and knowledge of nutrition (*Coefficient*: 5.050, 95% *CI*: 0.842–9.258) had a significantly positive impact on the intake duration.

**Table 5:** Results of multiple linear regression analyses of variables related to the duration of intake.

**Dependent variable: intake duration**	**(1)**		**(2)**		**(3)**	
	**Coefficient (95% CI)**	***P*** **-value**	**Coefficient (95% CI)**	***P*** **-value**	**Coefficient (95% CI)**	***P*** **-value**
**Knowledge**						
Overall knowledge of health and nutrition (standardized score)	9.278 (2.966–15.591)	0.004				
Overall knowledge of nutrition (standardized score)			5.050 (0.842–9.258)	0.019		
Overall knowledge of health (standardized score)					5.362 (0.465–10.259)	0.032
Knew correct time to take folic acid (1 = yes)	1.375 (−13.428–16.177)	0.855	2.074 (−13.046–17.194)	0.788	7.392 (−6.451–21.235)	0.295
Awareness of folic acid policy (1 = yes)	16.451 (4.555–28.347)	0.007	16.641 (4.726–28.555)	0.006	16.700 (4.779–28.621)	0.006
**Characteristics of respondents**						
Age (< 28 as reference)	1.414 (−9.990–12.818)	0.808	1.708 (−9.711–13.127)	0.769	1.652 (−9.777–13.081)	0.777
Years of education (≤ 9 years as reference)	−2.893 (−14.827–9.041)	0.634	−1.443 (−13.279–10.394)	0.811	−0.355 (−12.036–11.326)	0.952
Employment status (1 = employed)	8.606 (−3.619–20.832)	0.167	8.900 (−3.343–21.142)	0.154	9.024 (−3.223–21.271)	0.148
Pregnancy order (1 = first)	31.975 (19.856–44.095)	<0.001	32.402 (20.267–44.536)	<0.001	32.047 (19.895–44.199)	<0.001
Pregnancy intention (1 = intended)	29.273 (18.256–40.291)	<0.001	29.066 (18.032–40.101)	<0.001	29.167 (18.124–40.210)	<0.001
BMI (BMI < 25 as reference)						
25 ≤ BMI < 30	14.182 (2.788–25.576)	0.015	14.697 (3.275–26.119)	0.012	13.640 (2.208–25.072)	0.019
BMI ≥ 30	24.348 (7.459–41.237)	0.005	24.115 (7.198–41.032)	0.005	23.935 (7.011–40.858)	0.006
Self-reported health status (1 = excellent)	−5.282 (−16.952–6.389)	0.375	−4.549 (−16.244–7.146)	0.445	−5.834 (−17.555–5.887)	0.329
Living together with husband (1 = yes)	−12.599 (−23.799–1.400)	0.028	−12.502 (-23.721–−1.283)	0.029	−12.659 (−23.886–1.433)	0.027
Family asset in lowest 25% (1 = yes)	−6.768 (−19.480–5.943)	0.296	−7.843 (−20.520–4.833)	0.225	−7.527 (−20.251–5.198)	0.246
Officially registered as poverty-stricken household (1 = yes)	−5.015 (−20.185–10.155)	0.517	−5.247 (−20.441–9.947)	0.498	−5.356 (−20.557–9.846)	0.489
Region (1 = north)	21.486 (10.792–32.181)	<0.001	22.107 (11.369–32.844)	<0.001	20.616 (9.884–31.349)	<0.001

95% CI, 95% confidence intervals.

Besides, a longer duration of folic acid intake can be observed in expectant women who were aware of the folic acid policy, expecting for the first time, pregnant by intention, with higher BMI, and located in the northern areas. For women living together with husbands, the duration of folic acid intake was at least 12.5 days less than those who were not living together with their husbands.

## 4. Discussion

In this paper, we presented the current picking up rate and intake rate of folic acid supplements in rural areas of northwestern China, and investigated the effect of related knowledge, in terms of overall nutrition and health knowledge, knowledge of the accurate time of taking folic acid, as well as awareness of folic acid distribution policy. Results of our study indicate that lack of policy awareness was the major factor related to the low picking up and intake rate of free folic acid supplements, while the intake of folic acid can be also affected by pregnancy order and intention. Moreover, we also found that advanced knowledge of nutrition and health is associated with a longer duration of folic acid intake but had no significant impact on the picking up and intake of folic acid before pregnancy.

Compared with the high picking-up rate of free folic acid supplements, the actual usage of folic acid is less than optimal. 76.4% of subjects had picked up free folic acid distributed by the government, which is consistent with most previous studies conducted in Shaanxi and other rural areas (21, 22). However, similar to the situation in other countries (28, 30, 34), the actual usage of folic acid among our sample was only 44.5%. Moreover, our findings indicate that only 20.7% of our sample have taken folic acid supplements for the duration recommended by the Chinese National Health Commission (180 days in total) (20). We would then argue that the free mass distribution of folic acid does not necessarily equal effective utilization, and in fact, almost half of the folic acid supplements distributed to expectant mothers in rural Shaanxi were wasted. Some behavior economists suggest that according to the theory of loss aversion, people are more sensitive to losses than gains (35) so they would accept the free folic acid supplements. But according to the psychological sunk cost effect, people would cherish less to goods free of charge so the actual usage rate was low (36). However, the free folic acid supplements in our study were not always ineffective, especially for families expecting their first baby or the ones pregnant intentionally. Our results also found that the sample expectant mothers' knowledge instead of their economic status might be associated with the usage of folic acid supplements in some way.

As our results indicated, the aspects of knowledge were associated with the picking up and intake situation of folic acid supplements in different ways. In short, whether the expectant mothers pick up the free folic acid supplements mainly depends on whether they were aware of the policy. But the duration of usage was influenced by their knowledge level of health and nutrition. The majority of the women (71.7%) obtained their information on folic acid from doctors and staff of family planning; and as the Chinese government offers five free prenatal care to pregnant women, they can get information about folic acid during regular prenatal care (31). Therefore, women who were aware of the policy were more likely to also get counseling before conception and know the importance of taking folic acid periconceptionally. Nevertheless, despite the fact that the free folic acid distribution policy of China has been implemented for more than 10 years, there were still up to one-third (26.7%) of our subjects reported that they were not aware of the free folic acid distribution policy before the survey. Therefore, more efforts to strengthen policy advocacy are needed.

Considering the neural tube closes by day 28 of pregnancy (37), we also examined factors associated with periconceptional folic acid intake which is necessary for NTDs prevention. In line with previous studies (14), our study also suggested that planned pregnancy and pregnancy order were associated with periconceptional intake. This is because women who had planned their pregnancy would have the opportunity to consult with doctors and get more information to be prepared for the pregnancy both physically and mentally, while the positive association between the first pregnancy and periconceptional intake of folic acid can be explained by the fact that most first time mothers would act more precautious and willing to follow instructions on healthcare than women who were pregnant before.

Our results also showed that advanced knowledge of nutrition and health is associated with a longer duration of folic acid intake before and at early conception. This could be attributed to the fact that women with more knowledge of health and nutrition during pregnancy may pay more attention to their nutrient intake and be more compliant with the suggested duration of folic acid intake. In line with previous literature, other influencing factors of adequate folic acid intake included awareness of folic acid policy (22), order of pregnancy (19), pregnancy intention (38), BMI, whether living together with husband (39) and region (22).

Currently, although most Chinese women of reproductive age in rural areas were aware of the correct time to use folic acid and have access to free folic acid supplements, there is still a large gap between the “knowing” and “doing.” Pre-pregnancy healthcare and counseling services for rural women should be provided along with free folic acid supplements to ensure adequate folic acid usage before and during pregnancy.

Although the study does have important implications, it is not without limitations. First, the sample of our study was from rural areas of Shaanxi, China, so the results would be difficult to be generalized to other geographic regions. Second, 31.2% of the subjects reported that they have also purchased compound vitamins containing folic acid for themselves. But as we lack information about what kind of compound vitamins they purchased and the specific duration of intake, we were not able to figure out the association between the knowledge and intake of folic acid supplements purchased by pregnant women themselves. Third, since the data related to the picking up and usage of folic acid supplements were based on participants' retrospective reports, there might be potential recall bias which influenced the accuracy of measurement.

## 5. Conclusion

In sum, our study results indicated that compared with picking up rates of free folic acid supplements, folic acid intake was not ideal among pregnant women in rural areas of China. Folic acid policy awareness was positively associated with the picking up and intake of folic acid before and during conception. Knowledge of health and nutrition about pregnancy was related to more days of folic acid intake and had no impact on the picking up and intake of folic acid before pregnancy.

## Data availability statement

The raw data supporting the conclusions of this article will be made available by the authors, without undue reservation.

## Ethics statement

The studies involving human participants were reviewed and approved by Medical Ethics Committee of Shaanxi Normal University (Xi'an, China) and Xi'an Jiaotong University (Xi'an, China, No. 2020-1240). Written informed consent for participation was not required for this study in accordance with the national legislation and the institutional requirements.

## Author contributions

JYa and JN conceived the study. JYa, NW, and JN designed the questionnaires. ZR performed the analyses and interpreted the results with assistance from JYa and JN. JYa, ZR, YLiu, and YW drafted the manuscript. All authors critically revised the manuscript and approved the final manuscript as submitted.
